# Differential Effects of Parietal and Cerebellar Stroke in Response to Object Location Perturbation

**DOI:** 10.3389/fnhum.2015.00293

**Published:** 2015-07-13

**Authors:** Trudy A. Pelton, Alan M. Wing, Dagmar Fraser, Paulette van Vliet

**Affiliations:** ^1^School of Psychology, College of Life and Environmental Sciences, University of Birmingham, Edgbaston, UK; ^2^School of Health Sciences, Faculty of Health and Medicine, The University of Newcastle, Callaghan, NSW, Australia; ^3^Hunter Medical Research Institute, The University of Newcastle, Callaghan, NSW, Australia

**Keywords:** stroke, upper limb, coordination, arm, hand

## Abstract

**Background:**

The differential contributions of the cerebellum and parietal lobe to coordination between hand transport and hand shaping to an object have not been clearly identified.

**Objective:**

To contrast impairments in reach-to-grasp coordination, in response to object location perturbation, in patients with right parietal and cerebellar lesions, in order to further elucidate the role of each area in reach-to-grasp coordination.

**Method:**

A two-factor design with one between subject factor (right parietal stroke; cerebellar stroke; controls) and one within subject factor (presence or absence of object location perturbation) examined correction processes used to maintain coordination between transport-to-grasp in the presence of perturbation. Sixteen chronic stroke participants (eight with right parietal lesions and eight with cerebellar lesions) were matched in age (mean = 61 years; standard deviation = 12) and hand dominance with 16 healthy controls. Hand and arm movements were recorded during unperturbed baseline trials (10) and unpredictable trials (60) in which the target was displaced to the left (10) or right (10) or remained fixed (40).

**Results:**

Cerebellar patients had a slowed response to perturbation with anticipatory hand opening, an increased number of aperture peaks and disruption to temporal coordination, and greater variability. Parietal participants also exhibited slowed movements, with increased number of aperture peaks, but in addition, increased the number of velocity peaks and had a longer wrist path trajectory due to difficulties planning the new transport goal and thus relying more on feedback control.

**Conclusion:**

Patients with parietal or cerebellar lesions showed some similar and some contrasting deficits. The cerebellum was more dominant in controlling temporal coupling between transport and grasp components, and the parietal area was more concerned with using sensation to relate arm and hand state to target position.

## Introduction

Successful control of reach-to-grasp requires coordination, “an ability to maintain a context-dependent and phase-dependent cyclical relationship between different body segments or joints in both spatial and temporal domains” (Krasovsky and Levin, 2010) of various body segments including the arm with the trunk, the shoulder with the elbow, and the hand with the arm. Studies in healthy adults have suggested hand and arm function are controlled as a single coordinated unit (Jeannerod, 1984; Wallace et al., 1990) demonstrated by significant correlations between reach and grasp components, including between the start time of the opening of the hand and the start time of hand movement toward the object (Jeannerod and Biguer, 1982; Jeannerod, 1984), between the time of maximum hand aperture and the time of peak deceleration (PD) of the hand (Jeannerod, 1984; Castiello et al., 1993), and between time of maximum aperture (TMA) and the time of peak velocity (TPV) of the hand (Wallace et al., 1990). Apart from a few situations (Gentilucci et al., 1991; Kudoh et al., 1997), for example, where correlations between the time of PD and the time of MA are not reliable when transport and grasp were manipulated by the distance and type of grasp (Gentilucci et al., 1991), temporal coupling of these events is a fairly consistent finding across reach-to-grasp tasks.

Stroke can adversely affect reach-to-grasp coordination (Pelton et al., 2012; vanVliet et al., 2013). Spatiotemporal relationships between transport and grasp in a heterogenous group of stroke patients with mild to moderate impairments, were investigated in a study where movements were performed at both fast and preferred speeds and to small and larger objects (vanVliet and Sheridan, 2007). There were significant correlations (*p* < 0.05) between times of start of hand movement and hand opening and between times of MA and PD in all conditions for both groups, although some of the correlations were numerically small (Pearson product-moment correlation coefficient *r* for the two groups ranged from 0.3 to 0.71). However, transport and grasp in patients were not as tightly coupled. In the condition which most challenged accuracy (i.e., the fast paced condition with small objects), the two events were less correlated in participants with stroke.

One informative paradigm used to investigate underlying control mechanisms for coordination of reach-to-grasp is to perturb the object location at movement onset in order to examine the resulting temporal adjustments made to the grasp component (Paulignan et al., 1990, 1991a,b), requiring modification of a pre-defined program (Goodale et al., 1986). Typically, the unexpected perturbation in the object produced adjustments to both the transport and grasp components, where the initial wrist acceleration was aborted and a new one started, and the initial grasp aperture was also aborted and reincreased in synchrony (Paulignan et al., 1990, 1991a,b) demonstrating that the two components are coordinated spatiotemporally.

The premise of a tight-coupling between the two components prompted the development of a model for the temporal coordination of transport and grasp (Hoff and Arbib, 1993). The model proposed that neural processes controlling transport and grasp are monitored on-line and adjusted for temporally so that the expected duration of each trajectory to reach the target is matched to the other component according to a consistent enclose time of the hand. The coordinated control of transport-to-grasp with object location perturbation also involves the integration of sensory signals from multiple modalities (principally visual information concerning the object and its relative position; and proprioceptive information about the position of the arm and the hand). It requires the feed-forward selection of perhaps one or two coupled motor commands for transport and grasp together with a forward representation of the desired movement. Smooth movement is dependent upon on-line updating of the initial pattern of muscle activation and detection of error between the actual positions of object relative to the hand. Large errors which are instigated by the introduction of a perturbation require either rapid modification of an ongoing internal forward model or rapid onset of a new internal forward model and cessation of the old forward model.

Two key brain areas responsible for processing of information pertaining to reach-to-grasp coordination are the parietal lobe and the cerebellum. Current theories attribute similar contributions by parietal and cerebellar regions. For example, both areas have been identified as potential areas that integrate the independent motor processes for reach and grasp into one common motor program (Desmurget et al., 1999; Zackowski et al., 2002). Given the specialization of cortical areas, it is unlikely that these areas perform identical roles. Thus, further research is needed to elucidate their exact role in control of reach-to-grasp. It has been pointed out that the two regions may work as a functional loop in estimating the current status of the motor system, since the parietal cortex receives input from the cerebellar dentate nucleus, and there are connections from parietal cortex to cerebellum via the pontine nuclei (Blakemore and Sirigu, 2003; Ramnani, 2012). Below, we review the current knowledge about roles of parietal cortex and cerebellum in control of reach-to-grasp.

### Role of parietal cortex in control of reach-to-grasp

Two neural circuits that contribute to the control of coordination of transport and grasp between the parietal lobe and the premotor cortex have been identified in primates. For proximal muscles involved in transport, a medial circuit is described, which is concerned with object location. The medial circuit is associated with areas of the superior parietal lobule [area “MIP”/PRR (parietal reach region)] and the dorsal premotor Brodmann area 6. For the distal musculature involved in grasping, a lateral circuit is described which is concerned with the size and shape of the object. The lateral circuit is associated with the inferior parietal lobule (in particular the anterior intraparietal area) and the ventral premotor area 6 (Fattori et al., 2009). Overlap exists so that both circuits are partially involved in both processes and the dorsomedial pathway contributes to the integration of the two components (Fattori et al., 2010; Vesia and Crawford, 2012).

Parietal cortex is known to have a distinct role in processing sensory information. A recent review (Buneo et al., 2002) indicates that the posterior parietal cortex (PPC) plays a role in converting sensory information into motor commands and also for integrating sensory input with previous and ongoing motor commands to maintain a continuous estimate of arm state that can be used to update present and future movement plans. Decomposition of movement may therefore be the result of abnormal on-line processing of sensory information such as proprioception (Shimansky et al., 1997). Results from studies using transcranial magnetic stimulation have suggested the PPC is involved in computing current motor error to allow updating of muscle activation patterns (Desmurget et al., 1999; Tunik et al., 2005). The parietal cortex appears to play a dual role in feed-forward and feedback control of reach-to-grasp; transforming visual information into a motor plan (Vesia and Crawford, 2012) and making on-line corrections according to visual feedback (Iacoboni, 2006).

The PPC has specialized areas for spatial monitoring during reaching. The parietal reach region neurons within the PPC are selectively activated during reaches and are thought to encode target location (Batista and Andersen, 2001). Further, internal spatial monitoring is lost in monkeys with lesions involving the PPC, area 7 (Batista and Andersen, 2001). Humans with lesions in the intraparietal sulcus, superior parietal lobule, or inferior parietal lobule of the PPC also show directional errors in reaching (Karnath and Perenin, 2005). Control of the pre-shaping of grasp is thought to be located within the anterior intraparietal sulcus in the PPC (Binkofsky et al., 1998). A larger than normal MA has also been found after a posterior parietal lesion (Jeannerod, 1986).

Patients with lesions in the superior parietal lobe and adjacent intraparietal sulcus commonly also demonstrate optic ataxia, where mistransporting (missing the target) occurs to peripheral vision targets whilst maintaining a central fixation point (Jakobson et al., 1991; Wolpert et al., 1998). The parietal area also is thought to generate a representation of visual space based on retinal coordinates, which enables it to be involved in the planning of eye, reaching, and grasping movements (Blakemore and Sirigu, 2003).

### Role of cerebellum in control of reach-to-grasp

It has been proposed that the cerebellum provides an internal state estimate or sensory prediction used for the on-line control of movements (Miall, 1998; Ebner and Pasalar, 2008; Miall and King, 2008). These predictive state estimates are used to coordinate actions by the different effectors including the eye, the hand, and the arm (Miall and Wolpert, 1996) and to adjust the relative strength and timing of muscle activations based upon internal predictions about the likely outcome of the effector movement (Miall et al., 1993; Wolpert et al., 1998). The cerebellum is also important for making rapid adjustments to perturbations by modifying automatic movements that are dependent upon visual sensory information (Donchin and Rabe, 2012).

Deficits in transport and grasp have been shown in the hand ipsilateral to the lesion, tested in six patients with cerebellar lesions (Rand et al., 2000) where several velocity peaks were present, grip aperture was larger and more variable than normal. Transport deficits were also demonstrated in a trial of pointing movements (Topka et al., 1998), where patients showed more variable endpoints, and longer movement durations (MDs), with lower peak hand acceleration and deceleration and a longer deceleration, compared to control subjects and these deficits were accentuated in fast movements. People with cerebellar degeneration (resulting from spinocerebellar ataxia, sporadic adult onset ataxia, or autosomal dominant ataxia) have demonstrated slower movements, a more deviant trajectory and a larger hand aperture (Brandauer et al., 2008).

The cerebellum also plays an integral role in visuomotor adaptation. For example, patients with focal cerebellar lesions showed impaired motor learning of a cursor movement task with the handle of a robot when a proportion of the trials involved perturbations to either the visual rotation of the cursor or a force field of the manipulandum (Donchin and Rabe, 2012). Similarly, the path traveled during pointing is less efficient than in control subjects following on-line correction of movements in response to perturbation (Tseng et al., 2007). Furthermore, patients with cerebellar lesions show that reaching path corrections based upon visual sensory information are characterized by excessive deviations and abnormal oscillations (Day et al., 1998).

### Areas of similarity with regard to reach-to-grasp coordination in parietal and cerebellar regions

#### Combining the Two Components of Transport and Grasp into a Single Functional Unit

Both areas have been suggested as having a role in combining the two components of transport and grasp into a single functional unit. This is demonstrated by a PET study comparing separate finger and arm movements with a task in which both finger and arm movements were coordinated (to extend the finger when the arm passed a stationary target), revealing additional cortical activation for the coordination task in both parietal (intraparietal sulcus and medial PPC) and cerebellar (anterior lobe and paramedian lobules) areas (Ramnani et al., 2001). This suggests a role in coordinating arm and finger movements for both areas but does not reveal the exact role played by each. The involvement of both areas was hypothesized to be partly due to processing of proprioceptive information, given that the cerebellum receives major proprioception afferents from spinocerebellar tracts, and the parietal cortex is known to process information about arm position. The greater activation in the parietal cortex was also attributed to the task of localizing visual target position (Ramnani et al., 2001).

Several studies evidence a role for the parietal lobe in coordination of reach-to-grasp movements. Right inferior parietal lobe damage in one patient caused an inability to make multi-component movements with the left hand in the absence of visual feedback, whereas single component movements could be performed (Jeannerod et al., 1984). In a single case study, a lesion in the dorsal PPC caused a lack of the usual change in velocity of hand opening according to object size (Roy et al., 2004). In 32 patients with parietal stroke, abnormal anticipatory hand shaping and dysmetria were present (Ghika et al., 1998). Binkofsky et al. (1998) described the deficits of six subjects with stroke affecting the lateral bank of the anterior intraparietal sulcus in the PPC. These patients showed poor pre-shaping of the hand in the acceleration phase, increased and more variable aperture in the deceleration phase, and a later maximum grasp aperture as a percentage of MD, compared to control subjects, but almost normal velocity profiles. Because humans with PPC lesions also show directional errors in reaching (Karnath and Perenin, 2005), it has been suggested that the PPC has a role in coordinating arm and hand (Mackay and Riehle, 1992).

Similarly, there is evidence for a cerebellar role in reach-to-grasp coordination. In one group with cerebellar lesions, maximum grasp aperture (expressed as a percentage of MD) occurred earlier and was larger than for control subjects and the time from PV to time to MA was significantly smaller and more variable (Rand et al., 2000). Distinct deficits have been found in the temporal coupling of the transport and grasp components in cerebellar subjects (Zackowski et al., 2002) who showed within and between subject variability of time to MA, and a larger size and more aperture peaks compared to control subjects, which respectively could suggest compensation and correction for inaccurate transport of the hand. The changed relationship between transport and grasp may be the result of impaired parallel processing between the shoulder, elbow, and hand (Timman et al., 1999), which is normally controlled by the cerebellum.

#### Making On-line Adjustments in Response to Perturbation

Both brain areas have also been implicated in and making on-line adjustments in response to perturbations. Transport goals are represented in the parietal region and may have to be rapidly switched in response to perturbation (Snyder et al., 2000). On-line corrections during target errors depend upon the integrity of the PPC (Desmurget et al., 1999). Pisella et al. (2000) provide case study evidence in a stroke patient for the involvement of the parietal lobe in rapid modifications of pointing movements. It was observed that whilst unperturbed movements were normal in a patient with bilateral PPC lesions, when the target jumped to another position, her movements were slow and deliberate. Also, in the reach-to-grasp performance of the patient with a lesion of dorsal PPC, the normal adjustment of time to PV of the hand in response to change in movement direction was absent (Roy et al., 2004).

The cerebellum has a role in the updating of aimed movements. For example, Fisher et al. (2006) found that cerebellar subjects had errors in target direction and amplitude specification, despite ample preparation time, whereas final position was minimally impaired suggesting preserved ability to adapt or update the movements. Cerebellar damage would then likely place greater reliance upon on-line correction which would result in a longer deceleration phase. Evidence that the cerebellum plays a part in updating goal-directed movements is provided in one study where, unlike control subjects, patients with superior cerebellar artery infarctions could not accurately reach a target following perturbation during a visuomotor task (Donchin and Rabe, 2012).

### Aims and hypotheses

Current knowledge draws on studies conducted separately with patients with parietal or cerebellar damage, using different experimental paradigms. We aim to better understand the contribution of parietal and cerebellar regions to reach-to-grasp coordination, by testing the response of these two groups of patients to perturbation of object location in a common experimental paradigm.

Specific aims were (1) to identify specific reach-to-grasp coordination impairments associated with either parietal or cerebellar lesions and (2) to quantify how patients with right parietal or cerebellar lesions adjust transport-to-grasp when object location is perturbed. The object location paradigm as reported by Paulignan et al. (1991b) was used.

Key hypotheses regarding the stroke groups were that (a) right parietal lobe patients would demonstrate difficulties related to planning the new transport goal and reflect their reliance on feedback driven control, such as longer MD and more wrist velocity peaks; and (b) in contrast, cerebellar patients would show impaired adjustment of grasp aperture size in response to the object location perturbation.

## Materials and Methods

### Design

A factorial experimental design was employed, with three groups of participants – parietal, cerebellar, healthy controls, and two conditions – presence or absence of perturbation. Data collection took place in a movement laboratory, and was conducted in a single session.

### Participants

Sixteen participants with stroke were recruited consecutively from six hospitals and from a local research database of stroke patients. Inclusion criteria were: (1) cerebellar or right parietal stroke of ischemic or hemorrhagic origin, confirmed by CT scan, (2) a score of 6 or more on the arm section of the Rivermead Motor Assessment (RMA), i.e., transport forward, pick up tennis ball, release at mid-thigh on affected side × 5, (3) informed consent. Right-sided stroke was chosen because the right hemisphere has a preferential role for processing hand position, object location (Tretriluxana et al., 2009), and visual feedback for movement adjustments (Winstein and Pohl, 1995) compared to the left hemisphere. Exclusion criteria: (1) cognitive dysfunction preventing understanding of the task, (2) concurrent medical problems which prevent repetitive arm movement (e.g., shoulder pain). Clinical examination was undertaken by the research physiotherapist (first author TP) and included Fugl-Meyer Upper Extremity Motor Function (Fugl-Meyer et al., 1975); Revised Nottingham Sensory Assessment (NSA) (Lincoln et al., 1998); Nottingham Extended Activities of Daily Living (NEADL) (Nouri and Lincoln, 1987); classical testing procedures for tactile extinction (light touch with fingers to the subjects hand) (Tucker and Bigler, 1989) and visual extinction (in which the patient fixates the examiner’s nose, the examiner’s arms are outstretched, and the patient has to detect movements of the examiner’s index finger on either or both sides) (Baylis et al., 1993); Modified Ashworth Scale (MAS) (Bohannon and Smith, 1987); and the Medical Research Council scale (MRC strength test of the more involved upper limb) (Compston, 2010). Thirty control participants, matched to the stroke group according to age, gender, and hand dominance were also recruited. Stroke participants and controls were also assessed for the time taken to complete the 10 Hole Peg Test (10HPT) (Turton and Fraser, 1986). Informed consent was obtained from all participants and the study protocol was approved by the South Birmingham Research Ethics Committee (08/H1207/332).

### Protocol

**Figure 1:**
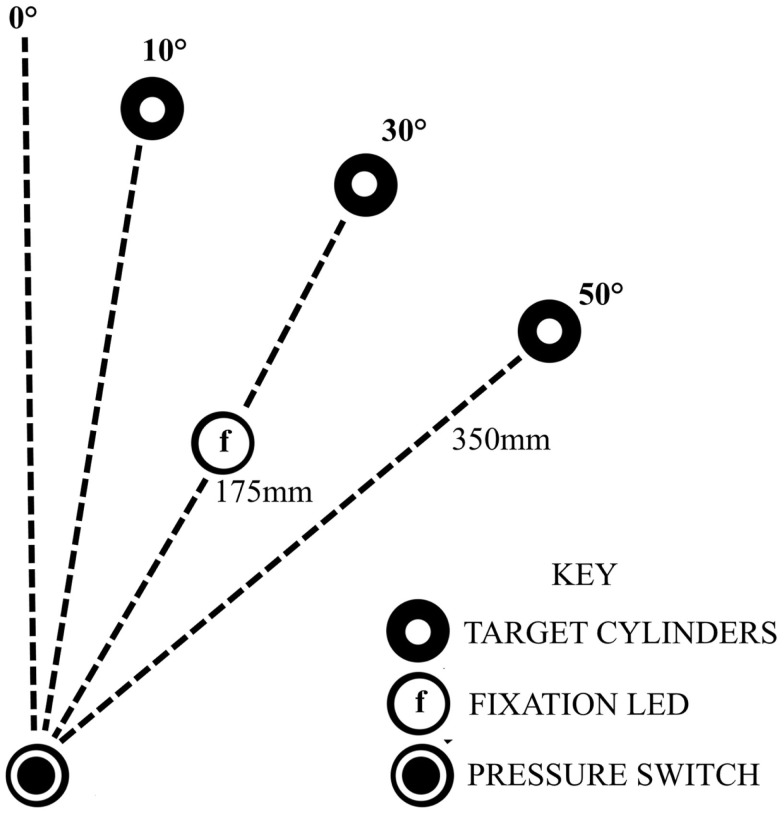
**Experimental set-up, showing the configuration for a participant with a ride sided lesion, reaching with affected left arm to the opposing side**.

Seated close to a table edge, participants were instructed to perform fast, accurate, reach-to-grasp movements with the more affected arm using a precision grip between the thumb and index finger. Participants were instructed to lift the object 2–4 cm off the table before replacing it in the approximate same position. The start position of the hand was resting on a pressure-sensitive switch close to the body in the mid-saggital axis, with the elbow flexed to approximately 90°, the forearm in mid-pronation and the pads of the index finger and thumb touching. Reach-to-grasp movements were to perspex cylinders (10 cm height × 1.5 cm in diameter), in three locations: 10°, 30°, or 50° to the opposing side of midline, each 35 cm from the start position (Figure 1). Following 6 practice trials and 10 unperturbed control trials, two blocks of 30 experimental trials ensued with a 5 min rest period between the two blocks. Each block consisted of a randomized sequence of 20 unperturbed trials to the 30° cylinder, 5 trials perturbed to the 10° cylinder, and 5 trials perturbed to the 50° cylinder. A different randomized sequence was performed by each stroke participant. Healthy participants performed the same randomized sequence as their matched stroke participant. A visual fixation light indicated the start of each trial. Participants were instructed to move as soon as they saw the illumination of the 30° object which occurred at a random time ranging between 500 and 2000 ms after the start of each trial. In perturbed trials, the perturbation occurred at movement onset by illumination of the 10° or 50° cylinders, via release of the start switch under the hand.

### Data acquisition

Data were captured using a Qualysys ProReflex MCU240 3D (Qualysis, 2006) motion analysis system with four infrared cameras and a sample rate of 200 Hz. The operation was controlled by an external trigger in MatLab and the data processed using Qualisys Track Manager software. Two cameras were positioned above the table and two in front. Data from reflective markers on the wrist (radial styloid process), the index finger nail, the thumb nail, and the sternal notch was analyzed using custom MatLab programs. Data were filtered using a Butterworth zero-phase forward and reverse digital filter with a cut-off of 8 Hz. Trajectory, velocity, and acceleration were calculated from the three dimensional coordinates of each marker.

### Measures

Reaction time (RT) was defined as the time (s) between the illumination onset of the 30° object and the wrist onset time [the time at which the wrist marker resultant velocity (across *x*, *y*, *z*) exceeded a threshold of 25 mm/s for five consecutive frames]. MD was characterized by the time (s) between wrist onset and object-lift-off (the time at which the velocity of the object exceeded 25 mm/s for five consecutive frames in the vertical *z* dimension). Grasp aperture was calculated as the maximum Euclidean distance between the markers on the thumb and index finger relative to the starting aperture distance. The maximum amplitude (millimeter) of grasp aperture was defined as the MA and the time (s) at which this occurred was recorded was defined as the TMA. The time at which the aperture velocity (differentiation of the distance between the finger and the thumb) exceeded 25 mm/s for five consecutive frames relative to the wrist start time was termed the aperture onset.

The wrist path trajectory (WPT) was defined as the sum of the three dimensional distance (millimeter) between each frame from wrist onset to object-lift-off. The absolute closure distance (CD) was calculated as the cumulative distance (millimeter) from MA to object-lift-off. CD was also expressed as a proportion of the total movement distance (CD %). Similarly, the trunk distance (TD) was calculated as the sum of the three dimensional distance (millimeter) traveled by the trunk marker between each frame from wrist onset to object-lift-off.

Peak wrist velocity (PV mm/s) referred to the absolute maximum amplitude of the tangential wrist velocity (derived from three dimensional WPT). PD (millimeter per second) was defined as minimum tangential wrist acceleration (derived from three dimensional WPT). The TPV and peak deceleration (TPD) occurred were expressed as absolute (s) and proportional (%MD) values.

Movement smoothness was quantified by the number of peaks in the tangential wrist velocity and the aperture size. Peaks were detected using a standard “Peakdet.m” (delta 0.5) MatLab file (Eli Billauer, 3.4.05) and counted if the difference between the peak and the preceding “valley” (minimum value) exceeded 15% of the global maximum amplitude (Kahn and Zygman, 2001). The number of identified wrist velocity and aperture size peaks was recorded. For each component, the absolute time of the last peak prior to object-lift-off was also recorded.

### Statistical analysis

The plan for statistical analysis was to (1) compare baseline characteristics of the stroke groups; (2) analyze differences between groups and conditions; (3) calculate correlation coefficients between transport and grasp events and compare these between groups; and (4) assess the relationship between the kinematic parameters and clinical impairment.

Unrelated sample *t*-tests were used to compare group characteristics such as age, time taken to complete the 10HPT, and time since stroke. Similarly, Mann–Whitney tests were performed on the scores for Fugl-Meyer UE motor function, NEADL, MRC muscle strength grading, and NSA. The number of participants in each patient group with tactile extinction, visual extinction, or reduced ROM was also recorded.

To analyze differences between groups and conditions, a two-way mixed ANOVA was used with group (control, parietal, cerebellar) as the between subject factor and condition (unperturbed and perturbed) as the within subject factor. *Post hoc* comparisons employed Bonferroni correction for multiple comparisons. Additionally, independent *t*-tests (*p* < 0.05) were performed to determine where significant differences originated as follows: effect of perturbation (perturbed vs. unperturbed trials) in parietal patients compared to controls; effect of perturbation in cerebellar patients compared to controls; effect of perturbation in parietal patients compared to cerebellar patients. Using the same analysis, the variability of the movements was compared between groups, indicated by the coefficient of variation (standard deviation divided by the mean of a set of trials).

Pearson product-moment correlation coefficients were used to examine the temporal relation between transport and grasp. Within-group correlation coefficients were calculated separately for each condition between the absolute time of maximum PV and the absolute time of maximum grasp aperture; the absolute time of PD and the absolute time of maximum grasp aperture; and finally between the absolute time of the last velocity peak and the last aperture peak (TLPA). Pearson’s *r* were later transformed to Fisher *z*: *z*_r_ = (1/2)[log_e_(1 + *r*) − log_e_(1 − *r*)] to test significance of *r* values and whether correlations differed between the stroke and control groups.

Fugl-Meyer scores (Fugl-Meyer et al., 1975) have previously been associated with a good prognosis for recovery after stroke (Prabhakaran and Zarahn, 2008). It was therefore considered of additional interest to observe the correlation between clinical impairment according to Fugl-Meyer scores and the RTG movement variables. This information may help to identify the most objective and sensitive variable for RTG function that could guide prognosis and measure performance. SPSS version 18 was used to perform the statistical analyses.

## Results

### Overview participant characteristics

**Figure 2:**
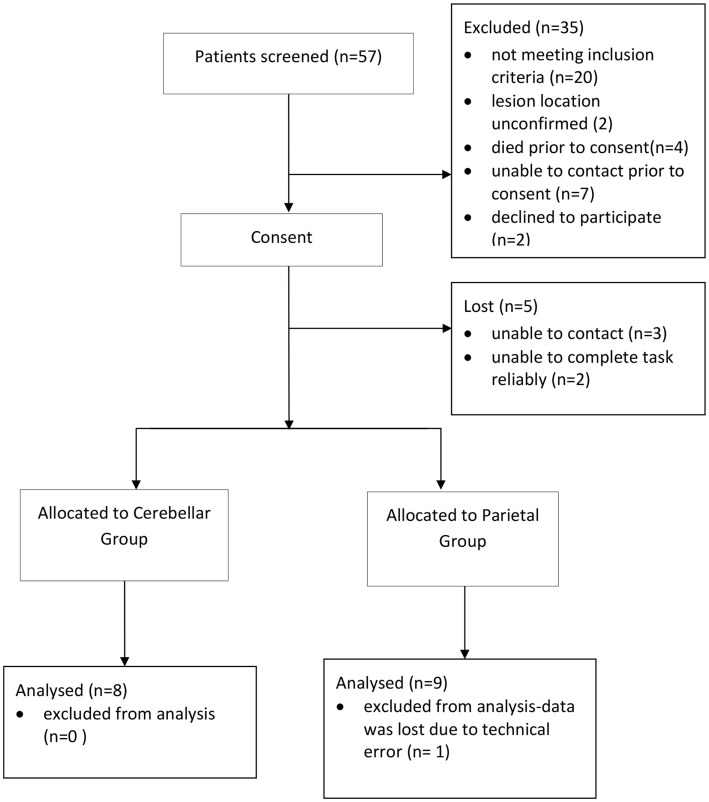
**Recruitment flow diagram**.

A total of 57 patients were screened and 16 patients (8 parietal and 8 cerebellar) were recruited (Figure 2). Due to difficulty recruiting participants with cerebellar stroke, the inclusion criteria were widened to include patients with cerebellar/pontine lesions. Unrelated sample *t*-tests revealed no statistical age differences between the control group (*N* = 16, *M* = 62 years, SE = 3), the parietal group (*N* = 8, *M* = 59 years, SE = 5), or the cerebellar group participants (*N* = 8, *M* = 62 years, SE = 4). Mean time to complete the 10HPT was significantly faster (*t*_13_ = 2.580, *p* < 0.05) for control participants (*M* = 12 s, SE = 0.5) than for the stroke patients (*M* = 27 s, SE = 6). Unrelated sample *t*-tests revealed no significant time difference for the 10HPT between the two patient groups (Table 1). Time since stroke was significantly longer for the parietal group than the cerebellar group (*t*_14_ = 3.002, *p* = 0.01). Mann–Whitney U-tests showed no statistical difference between patient groups in terms of the Fugl-Meyer, Extended Activities of Daily Living, and muscle strength tests. A significant difference (*p* < 0.01) in the NSA was found between the two patient groups; the parietal group demonstrated sensory impairment whereas for the cerebellar group sensation was intact. The parietal group included five participants with tactile extinction and two participants with visual extinction. There were no visual or tactile extinction problems identified in the cerebellar group.

**Table 1:** **Participant characteristics with mean (SD)**.

Group	Lesion	Time since stroke (months)	Age (years)	10 HPT (s)	NSA (9^a^)	NEADL (63^a^)	FMUL (66^a^)	Extinction		FROM (y/n)	MAS (5^a^)	Oxford muscle (5^a^)
								Tactile (y/n)	Visual (y/n)			
**PARIETAL**												
1	R P	25	55	52	5	48	48	n	n	y	0	4
2	R P-T	131	75	16	6	26	64	n	y	y	0	4
3	R F-P	22	62	33	6	40	55	y	n	y	0	5
4	P (bilateral)	24	33	19	0	33	55	y	n	y	0	5
5	R P	68	55	15	6	53	65	y	y	y	0	4
6	R P	48	61	104	2	33	45	y	n	n	2	4
7	R P	124	57	17	4	32	58	y	n	y	1	4
8	R P	30	72	14	9	41	62	n	n	y	2	4

*N* = 8		59 (45)	59 (13)	34 (31)	5 (3)	38 (9)	57 (7)	5	2	1	3	4.3 (0.5)
**CEREBELLAR**												
1	CP-A	24	60	14	9	63	63	n	n	y	0	5
2	R Cb	4	64	14	9	54	64	n	n	y	0	5
3	Pontine	12	66	19	9	53	44	n	n	n	2	4
4	R Cb	6	63	17	9	24	55	n	n	n	0	3
5	L Cb	3	61	18	9	46	64	n	n	y	0	5
6	L Cb	3	45	54	9	28	46	n	n	n	0	4
7	Cb (bilateral)	24	81	17	9	33	64	n	n	n	0	4
8	Cb (bilateral)	6	56	13	9	50	64	n	n	n	0	4

*N* = 8		10 (9)	62 (10)	21 (14)	9 (0)	44 (14)	58 (9)	0	0	2	1	4.3 (0.8)

*R, right; P, parietal; P-T, temporoparietal; F-P, frontoparietal; CP-A, cerebellar pontine angle; cb, cerebellar*.

*^a^Maximum possible score*.

*10 HPT (s) = time taken to complete. NSA (Nottingham Sensory Assessment score) for the hand consisting of – Light touch, pin prick, and stereognosis (0 = absent, 1 = impaired, 2 = normal), and proprioception [0 = absent, 1 = appreciation of movement (wrong direction), 2 = appreciation of direction of movement, 3 = normal]. NEADL (Nottingham Extended ADL index). Average from a maximum of 63 = 21 activities (0 = not at all, 1 = with help, 2 = alone with difficulty, 3 = alone easily). FMUL (Fugl-Meyer Assessment upper limb). Extinction (y = yes, n = no). FROM [Full active range of movement (y = yes, n = no)]. MAS (Modified Ashworth scale) (0 = no increase in muscle tone, 5 = rigid). Oxford Scale Muscle Strength (1 = flicker of movement, 5 = movement through range with full resistance)*.

First, the main effects and interactions of condition and group comparisons will be described. Secondly, measures of hand and arm coordination are presented in terms of the relationship between key events of transport and grasp. For simplification, data from the two groups of control participants were pooled. Means and standard deviations of kinematic parameters are summarized in Table 2. Significant interactions of group by condition are summarized in Table 3.

**Table 2:** **Means and standard errors (SE) for kinematic parameters in each group and each condition**.

	Controls Mean (SE)	Parietal Mean (SE)	Cerebellar Mean (SE)
**Movement onset (s)**			
Baseline	0.32 (0.03)	0.74 (0.14)	0.46 (0.04)
Unperturbed	0.38 (0.03)	0.71 (0.07)	0.63 (0.07)
Perturbed 10°	0.40 (0.05)	0.66 (0.07)	0.60 (0.21)
Perturbed 50°	0.35 (0.03)	0.69 (0.07)	0.66 (0.23)
**Movement duration (s)**			
Baseline	0.74 (0.05)	1.58 (0.39)	1.32 (0.25)
Unperturbed	0.77 (0.05)	1.62 (0.28)	1.30 (0.18)
Perturbed 10°	0.92 (0.08)	1.80 (0.28)	1.52 (0.18)
Perturbed 50°	0.88 (0.05)	1.86 (0.32)	1.40 (0.11)
**Peak wrist velocity (mm/s)**			
Baseline	889.00 (42.00)	605.00 (75.31)	553.00 (60.81)
Unperturbed	812.00 (33.50)	549.00 (68.94)	543.00 (43.84)
Perturbed 10°	787.00 (35.75)	534.00 (68.59)	496.00 (45.61)
Perturbed 50°	802.00 (37.00)	533.00 (65.05)	556.00 (38.54)
**Wrist path trajectory (mm)**			
Baseline	365.00 (8.75)	423.00 (34.65)	357.00 (16.26)
Unperturbed	365.00 (8.50)	404.00 (19.45)	361.00 (17.32)
Perturbed 10°	390.00 (12.50)	442.00 (18.74)	379.00 (24.75)
Perturbed 50°	414.00 (9.00)	460.00 (17.32)	404.00 (18.03)
**Absolute time to peak velocity (s)**			
Baseline	0.28 (0.02)	0.41 (0.05)	0.41 (0.06)
Unperturbed	0.29 (0.01)	0.48 (0.09)	0.41 (0.05)
Perturbed 10°	0.31 (0.03)	0.50 (0.13)	0.42 (0.08)
Perturbed 50°	0.30 (0.08)	0.56 (0.13)	0.44 (0.05)
**Normalized time to peak velocity (%)**			
Baseline	38.90 (2.20)	27.90 (3.29)	34.80 (2.72)
Unperturbed	39.00 (2.05)	30.50 (3.78)	33.80 (2.23)
Perturbed 10°	32.20 (1.45)	25.10 (3.68)	27.60 (3.36)
Perturbed 50°	34.30 (2.25)	27.70 (2.65)	31.70 (2.40)
**Absolute time of peak deceleration (s)**			
Baseline	0.42 (0.02)	0.77 (0.20)	0.66 (0.11)
Unperturbed	0.45 (0.02)	0.85 (0.21)	0.64 (0.08)
Perturbed 10°	0.48 (0.04)	0.80 (0.19)	0.65 (0.10)
Perturbed 50°	0.48 (0.02)	0.86 (0.19)	0.65 (0.05)
**Normalized time of peak deceleration (%)**			
Baseline	58.52 (4.94)	49.99 (4.03)	53.67 (3.32)
Unperturbed	60.30 (3.61)	52.00 (5.24)	51.86 (3.77)
Perturbed 10°	51.76 (2.31)	43.58 (4.42)	44.31 (3.97)
Perturbed 50°	55.13 (2.49)	46.53 (3.60)	47.56 (3.46)
**Grasp onset time (s)**			
Baseline	0.04 (0.02)	0.04 (0.04)	-0.04 (0.04)
Unperturbed	0.05 (0.02)	0.11 (0.05)	-0.04 (0.05)
Perturbed 10°	0.07 (0.04)	0.14 (0.05)	-0.01 (0.03)
Perturbed 50°	0.05 (0.03)	0.16 (0.06)	-0.05 (0.07)
**Maximum aperture (mm)**			
Baseline	59.80 (4.95)	59.70 (3.78)	59.50 (7.78)
Unperturbed	56.30 (5.00)	63.00 (5.41)	52.10 (6.12)
Perturbed 10°	59.00 (4.10)	69.40 (6.86)	55.40 (7.32)
Perturbed 50°	56.10 (4.38)	66.90 (6.01)	53.00 (7.42)
**Absolute time of maximum aperture (s)**			
Baseline	0.48 (0.03)	0.98 (0.20)	0.78 (0.11)
Unperturbed	0.54 (0.02)	1.07 (0.24)	0.83 (0.10)
Perturbed 10°	0.69 (0.04)	1.22 (0.47)	1.00 (0.11)
Perturbed 50°	0.63 (0.03)	1.22 (0.24)	0.90 (0.09)
**Normalized time of maximum aperture (%)**			
Baseline	65.90 (2.45)	64.20 (2.33)	64.10 (4.00)
Unperturbed	70.90 (2.38)	64.80 (5.87)	66.00 (4.28)
Perturbed 10°	75.00 (1.60)	70.50 (3.89)	67.60 (4.53)
Perturbed 50°	72.50 (2.25)	66.40 (5.16)	65.90 (4.74)
**Normalized closure distance (%)**			
Baseline	14.70 (1.60)	15.44 (3.03)	15.63 (2.07)
Unperturbed	12.44 (1.73)	19.33 (3.69)	17.30 (3.75)
Perturbed 10°	9.45 (0.94)	16.13 (4.47)	15.92 (4.46)
Perturbed 50°	10.54 (0.98)	15.25 (3.01)	14.59 (4.18)
**Aperture peaks (*n*)**			
Baseline	1.00 (0.00)	1.30 (0.14)	1.40 (0.11)
Unperturbed	1.10 (0.03)	1.20 (0.07)	1.10 (0.04)
Perturbed 10°	1.70 (0.10)	1.60 (0.14)	1.60 (0.11)
Perturbed 50°	1.70 (0.08)	1.60 (0.11)	1.50 (0.14)
**Velocity peaks (*n*)**			
Baseline	1.00 (0.00)	2.10 (0.67)	1.50 (0.25)
Unperturbed	1.00 (0.00)	2.10 (0.28)	1.60 (0.32)
Perturbed 10°	1.60 (0.10)	2.50 (0.57)	2.30 (0.35)
Perturbed 50°	1.70 (0.08)	2.70 (0.53)	2.00 (0.28)

**Table 3:** **Summary of significant interactions between groups and conditions**.

Kinematic parameter	Parietal vs. cerebellar		Parietal vs. control		Cerebellar vs. control	
	B/UP	10°/50°	B/UP	10°/50°	B/UP	10°/50°
Reaction time (s)			✓^↑^	✓^↑^	✓^↑^	✓^↑^
Movement duration			✓^↑^	✓^↑^	✓^↑^	✓^↑^
Peak wrist velocity (mm/s)			✓^↓^	✓^↓^	✓^↓^	✓^↓^
Wrist path trajectory (mm)		✓^↑^(50° only)	✓^↑^(U only)	✓^↑^		
Normalized time to peak velocity (%)			✓^↓^	✓^↓^		
Grasp onset time (s)	✓^↑^(U only)	✓^↑^				
Maximum aperture (mm)						
Normalized time to maximum aperture (%)						
Normalized closure distance (%)						
Aperture peaks (*n*)			✓^↑^(B only)		✓^↑^(B only)	
Velocity peaks (*n*)			✓^↑^	✓^↑^		✓^↑^(10° only)

*B/U, baseline and unperturbed condition; 10° and 50°, perturbed conditions; ✓, significant for both conditions in the column unless indicated otherwise. ^↑^ or ^↓^ indicates whether the kinematic parameter was increased, or decreased, in comparison to the control or cerebellar group*.

### Comparison between conditions

#### Transport Component

The four conditions consisted of (1) Baseline trials (the first 10 unperturbed trials), (2) Unperturbed trials to the 30° target, (3) Perturbed trials to the 10° target, and (4) Perturbed trials to the 50° target. As was expected, the RT was similar for each condition, whereas the average MD (*M* = 1.2 s, SE = 0.1) was longer (*F*_3, 87_ = 8.747, *p* < 0.01) in response to a perturbation in object location.

Peak wrist velocity showed a main effect for condition (*F*_3, 29_ = 7.592, *p* < 0.01). Overall, the PV during baseline trials (*M* = 682 mm/s, SE = 33.7) was higher (*p* < 0.05) than for all other conditions and for perturbed 10° trials (*M* = 606 mm/s, SE = 29) it was lower (*p* < 0.01) than the other conditions. The mean PV was however similar between the unperturbed (*M* = 635 mm/s, SE = 28) and perturbed 50° trials (*M* = 630 mm/s, SE = 28). Perturbation significantly (*F*_3, 29_ = 24.323, *p* < 0.01) increased the overall WPT. There was a significant difference (*p* < 0.01) in WPT between the two perturbed conditions (perturbed 10°*M* = 404 mm, SE = 10 and perturbed 50°*M* = 426 mm, SE = 8) but no difference between baseline (*M* = 382 mm, SE = 11) and unperturbed trials (*M* = 377 mm, SE = 8). The normalized time to PV (%TPV) was significantly different between conditions (*F*_3, 87_ = 8.447, *p* < 0.01). For perturbed 10° trials (*M* = 28%, SE = 2), %TPV was earlier than baseline (*M* = 34%, SE = 2, *p* < 0.05); unperturbed (*M* = 34%, SE = 2, *p* < 0.01) and perturbed 50° trials (*M* = 31%, SE = 1, *p* = 0.06).

It was assumed that in response to a perturbation in the object location there would be a normal adjustment to the wrist profile resulting in a second velocity peak. Indeed, we found a significant effect of condition (*F*_3, 87_ = 33.357, *p* < 0.01) with more wrist velocity peaks observed for perturbed 10° trials (*M* = 2.1, SE = 0.2) and perturbed 50° trials (*M* = 2.1, SE = 0.2) than for unperturbed (*M* = 1.5, SE = 0.2) and baseline trials (*M* = 1.6, SE = 0.2).

#### Grasp Component

The average distance between the thumb and finger markers at MA was 6 cm, for a 1.5 cm wide cylinder. As was expected, there was no main effect of condition on the aperture onset time. Perturbation of the object location had a significant effect upon the amplitude of the maximum grasp aperture (*F*_3, 87_ = 4.128, *p* < 0.01) and a significant condition and group interaction was observed (*F*_3,87_ = 3.360, *p* < 0.01). MA for perturbed 10° trials (*M* = 61 mm, SE = 3) and perturbed 50° trials (*M* = 59 mm, SE = 3) was larger (*p* < 0.01 and *p* < 0.05, respectively) than for unperturbed trials (*M* = 56 mm, SE = 3) and this difference was larger in the parietal group compared to the other two groups. For the normalized time to MA, TMA%, a significant main effect of condition (*F*_3, 87_ = 5.282, *p* < 0.05) was observed (Baseline *M* = 65%, SE = 2; Unperturbed *M* = 67%, SE = 3; Perturbed 10°*M* = 71%, SE = 2; Perturbed 50°*M* = 68%, SE = 2). MA occurred significantly (*p* < 0.01) later for perturbed 10° trials than at baseline.

There was a significant main effect of condition (*F*_3, 87_ = 31.508, *p* < 0.01) upon the number of aperture peaks suggesting that perturbation of transport resulted in the adjustment of the grasp aperture. Trunk movement was not significantly affected by the perturbation and there was no interaction effect upon the anterior trunk displacement. Both the absolute and the normalized CD were statistically similar for each condition.

### Comparisons between groups

#### Transport Component

There was a significant group difference in the RT (*F*_2, 29_ = 12.957, *p* < 0.01). RT was consistently longer for the parietal group (*M* = 0.7 s, SE = 0.1) in comparison to controls (*M* = 0.4 s, SE = 0.04) (baseline *t*_7_ = −3.321, *p* = 0.01; unperturbed *t*_22_ = −4.868, *p* < 0.01; perturbed 10°*t*_22_ = −2.856, *p* < 0.01 and perturbed 50°*t*_22_ = −2.856, *p* < 0.01). RT for the cerebellar group (*M* = 0.60 s, SE = 0.06) was similarly longer (baseline *t*_22_ = −3.316, *p* = 0.01; unperturbed *t*_22_ = −4.088, *p* < 0.01; perturbed 10°*t*_22_ = −2.387, *p* < 0.05 and perturbed 50°*t*_9_ = −4.046, *p* < 0.01). There was no statistical difference between the two patient groups. The variation in RT (*M* = 37, SE = 2.7) was similar between groups. A significant group difference in the mean MD was observed (*F*_2,29_ = 8.967, *p* < 0.01). As predicted, MD was shorter for control participants (*M* = 0.83 s, SE = 0.13) than for patients with parietal lesions (*M* = 1.72s, SE = 0.18) most reliably so after the baseline trials (baseline *t*_7_ = −2.238, *p* = 0.06; unperturbed *t*_7_ = −2.930, *p* < 0.05; perturbed 10°*t*_8_ = −3.156, *p* = 0.01 and perturbed 50°*t*_7_ = −2.983, *p* < 0.05). Comparisons between the cerebellar group (*M* = 1.39 s, SE = 0.18) and controls yielded similar differences (baseline *t*_7_ = −2.185, *p* = 0.06; unperturbed *t*_8_ = −2.872, *p* < 0.05; perturbed 10°*t*_9_ = −3.233, *p* = 0.01; and perturbed 50°*t*_10_ = −4.345, *p* < 0.01). There were no statistical differences between the two patient groups. The variation in MD was similar between groups.

The mean PV (*M* = 684 mm/s, SE = 37) was reduced significantly (*F*_2, 29_ = 13.795, *p* < 0.01) after parietal stroke. *M* = 555 mm/s, SE = 53 compared to controls *M* = 823 mm/s, SE = 37 in each condition (baseline *t*_22_ = 3.582, *p* < 0.01; unperturbed *t*_22_ = 3.907, *p* < 0.01; perturbed 10°*t*_22_ = 3.640, *p* < 0.01 and perturbed 50°*t*_22_ = 3.865, *p* < 0.01). For the cerebellar group, PV (*M* = 537 mm/s, SE = 53) was also consistently lower than controls (baseline *t*_22_ = 4.596, *p* < 0.01; unperturbed *t*_22_ = 4.764, *p* < 0.01; perturbed 10°*t*_22_ = 4.874, *p* < 0.01 and perturbed 50°*t*_22_ = 4.159, *p* < 0.01). There was no statistical difference between the two patient groups. We found no difference in variability of PV between groups.

**Figure 3:**
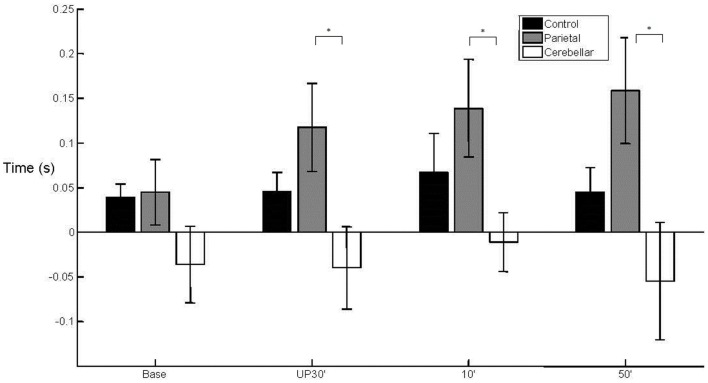
**Wrist path trajectory**. Mean (SE) effect of group (control, parietal, and cerebellar) and condition (baseline, unperturbed 30°, perturbed 10°, and perturbed 50°).

There was a between group difference (*F*_2, 29_ = 3.810, *p* < 0.05) in the mean WPT (*M* = 393 mm, SE = 9) (Figure 3) which was longer for the parietal group (*M* = 432 mm, SE = 16) than for the control group (baseline not significant; unperturbed *t*_22_ = −2.200, *p* < 0.05; perturbed 10°*t*_22_ = −2.330, *p* < 0.05 and perturbed 50°*t*_22_ = −2.648, *p* = 0.01). The WPT of the parietal group was also significantly longer than that of the cerebellar group, for the perturbed 50° trials (baseline not significant; unperturbed not significant; perturbed 10°*t*_14_ = −2.018, *p* = 0.06; perturbed 50°*t*_14_ = −2.234, *p* < 0.05). The mean WPT of the cerebellar group (*M* = 376, SE = 16) was more similar to that of controls (*M* = 383 mm, SE = 11). We found no group difference in the coefficient of variation for the WPT. For the normalized time to PV, there was a between group effect (*F*_2,29_ = 4.159, *p* < 0.05) with PV occurring comparatively early particularly in the parietal group, representing a prolonged deceleration phase (Controls *M* = 36%, SE = 2; Parietal *M* = 28%, SE = 2; Cerebellar *M* = 32%, SE = 2). %TPV occurred significantly earlier for the parietal group in comparison to controls (baseline *t*_22_ = −2.817, *p* = 0.01; unperturbed *t*_22_ = −2.145, *p* < 0.05; perturbed 10°*t*_22_ = −2.159, *p* < 0.05 and perturbed 50°*t*_22_ = −2.208, *p* < 0.05). In contrast, there were no differences between controls and the cerebellar group. We found no significant differences in %TPV between the two patient groups.

The mean number of wrist velocity peaks (*M* = 1.9, SE = 0.2) was higher (*F*_2, 29_ = 3.415, *p* < 0.05) after stroke (Controls *M* = 1.3, SE = 0.2; Parietals *M* = 2.4, SE = 0.3 and Cerebellar *M* = 1.9, SE = 0.3). Pairwise comparisons showed there to be significantly more wrist velocity peaks for the parietal group than for controls (*p* < 0.05). Comparisons between the controls and the cerebellar participants yielded a significant difference for the perturbed 10° trials only (*t*_22_ = −2.672, *p* < 0.05). There was no significant difference in the number of velocity peaks between the patient groups. The variability in the number of peaks was statistically similar between the three groups.

Anterior trunk displacement was higher for stroke participants however the group difference in amplitude was not significant (Controls *M* = 31 mm, SE = 10; Parietal *M* = 51 mm, SE = 14; and Cerebellar *M* = 68 mm, SE = 14).

#### Grasp Component

**Figure 4:**
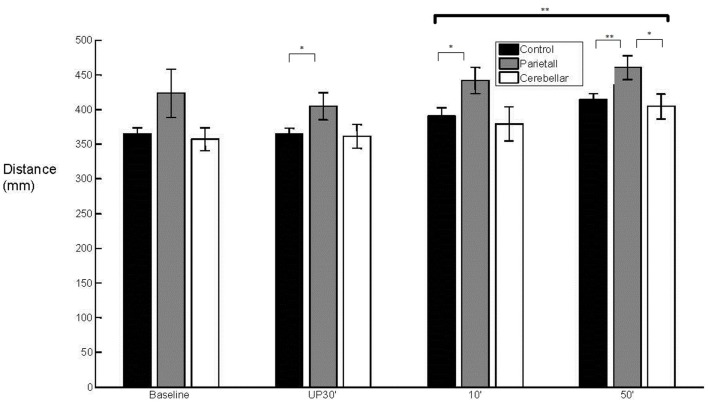
**50° aperture onset relative to wrist onset**. Mean (SE) effect of group (control, parietal, and cerebellar) and condition (baseline, unperturbed 30°, perturbed 10°, and perturbed 50°).

With regard to the grasp component, there was a main effect (*F*_2,29_ = 3.902, *p* < 0.05) of group upon aperture onset time (Figure 4). The cerebellar group demonstrated an early aperture onset (*M* = −0.036 s, SE = 0.038) whereas for the parietal group it was relatively late (*M* = 0.115 s, SE = 0.038). Following the baseline trials, significant differences were found between the two stroke groups (baseline NS; unperturbed *t*_14_ = −2.338, *p* < 0.05; perturbed 10°*t*_14_ = −2.351, *p* < 0.05 and perturbed 50°*t*_14_ = −2.413, *p* < 0.05). There were no significant differences for either of the patient groups in comparison to controls (*M* = 0.049 s, SE = 0.027). Overall, the mean MA was similar between the control group (*M* = 57 mm, SE = 4) and the stroke patient groups (Parietal *M* = 65 mm, SE = 6 and Cerebellar *M* = 55 mm, SE = 6). There was a tendency for increased variability of MA in the parietal group (*M* = 15%, SE = 2.1) when compared to controls (*M* = 10%, SE = 0.7) during the unperturbed trials only.

On average, TMA% (percentage time of MA) was comparable between stroke patients and controls (Controls *M* = 71%, SE = 2; Parietal *M* = 67%, SE = 3; Cerebellar *M* = 66%, SE = 3). The timing of TMA% was found to be more variable however in the parietal group in comparison to controls during baseline trials (*t*_22_ = −2.957, *p* < 0.01). There was also a tendency for more variability in the cerebellar group during perturbed 10° trials (*t*_22_ = −2.00, *p* = 0.058) in contrast to controls.

Whilst the normalized CD (Controls *M* = 12%, SE = 1; Parietal *M* = 17%, SE = 4; Cerebellar *M* = 16%, SE = 4) appeared longer and more variable in the stroke participants, this was statistically similar between groups.

Overall, the mean number of aperture peaks was comparable between stroke participants and controls (Controls *M* = 1.4 peaks, SE = 0.04; Parietal *M* = 1.4 peaks, SE = 0.06 and Cerebellar *M* = 1.4 peaks, SE = 0.06). A significant group and condition interaction (*F*_6, 87_ = 3.064, *p* < 0.01) was observed in the number of aperture peaks. During baseline trials, the parietal group (*M* = 1.3, SE = 0.13) and the cerebellar group (*M* = 1.4, SE = 0.32) showed a significantly greater number of aperture peaks (parietal baseline *t*_22_ = −2.864, *p* < 0.01 and cerebellar baseline *t*_22_ = −4.152, *p* < 0.01) in comparison to controls (*M* = 1.0, SE = 0.04). Following the baseline trials, the number of aperture peaks was similar for the three groups.

Since it was found the groups differ in time after stroke and sensation, groups were additionally split according to time since stroke (greater or less than 6 months post stroke), and sensation (a score of 9 or less than 9), and then an additional ANOVA was performed to examine the effect of these findings. There were no significant differences between groups in maximum grasp aperture, PV, RT, movement time, time to PV, time to MA, normalized time to PV and MA, or WPT, according to presence of sensory impairment. There was a significant effect of time since stroke upon WPT (*F*_1, 14_ = 8.312, *p* < 0.01) but there was no significant interaction between group and condition upon any of the kinematic variables.

### Coordination between key events of transport and grasp

#### Pearson Product-Moment Correlation Coefficients

Pearson product-moment correlation coefficients were used to determine if the absolute time to PV or absolute time to PD was correlated with the absolute time of maximum grasp aperture. The correlation coefficients were calculated separately within groups for perturbed and non-perturbed trials. Perturbations in object location usually elicited more than one peak in wrist velocity and aperture profiles. A further analysis was therefore performed using the PV and MA detected immediately prior to object-lift-off to determine the relationship between these more specific events (Table 4). For between group comparisons, *r* values were transformed to Fisher *z* values. The significance of the difference between *z* values was tested using two-way mixed ANOVAs with repeated measures and *post hoc* analysis (Table 4).

**Table 4:** **Pearson product-moment correlation r values and mean (SE) *z* scores, for correlations between (1) absolute times of peak velocity and maximum aperture; (2) absolute times of peak deceleration and maximum aperture; and (3) absolute times of the last peak velocity and the last maximum aperture**.

Group		Baseline			Unperturbed			Perturbed		
		PV and MA	PD and MA	Last PV and last MA	PV and MA	PD and MA	Last PV and last MA	PV and MA	PD and MA	Last PV and last MA
Control	*r*	0.83^a^	0.85^a^	0.47^a^	0.63^a^	0.65^a^	0.62^a^	0.78^a^	0.77^a^	0.86^a^
	*z*	1.02 (0.2)	1.06 (0.2)^b^	0.76 (0.1)	0.97 (0.2)	0.93 (0.1)	0.67 (0.1)	0.53 (0.2)	0.54 (0.1)	1.16 (0.2)^c^
Parietal	*r*	0.52^a^	0.81^a^	0.94^a^	0.65^a^	0.82^a^	0.86^a^	0.78^a^	0.83^a^	0.86^a^
	*z*	0.55 (0.2)	0.74 (0.2)^a^	0.47 (0.2)	0.86 (0.2)	0.75 (0.2)	0.80 (0.1)	0.80 (0.2)	0.68 (0.2)	1.19 (0.2)^c^
Cerebellar	*r*	0.84^a^	0.69^a^	0.81^a^	0.72^a^	0.49^a^	0.69^a^	0.61^a^	0.47^a^	0.63^a^
	*z*	1.02 (0.2)	0.67 (0.2)	0.87 (0.2)	0.65 (0.2)	0.55 (0.2)^d^	0.64 (0.1)	0.40 (0.2)	0.32 (0.2)	0.95 (0.2)^c^

*^a^Significant correlation (*p* < 0.01)*.

*^b^Significant effect of condition (*p* < 0.05) (baseline compared to other conditions)*.

*^c^Significant effect of condition (*p* < 0.05) (perturbed compared to other conditions)*.

*^d^Trend toward effect of group (cerebellar compared to other groups) (*p* < 0.06)*.

*PV, peak velocity; MA, maximum aperture; PD, peak deceleration*.

Although correlations between the temporal events were present in all groups and conditions, as summarized in Table 4, there were some differences. The correlations of PV and MA were less common in perturbed trials. For unperturbed trials, a significant correlation between the time of the PV and the time of MA was demonstrated by 88% of the control participants, 75% of the parietal group, and 63% of the cerebellar group. Whereas for perturbed trials, this correlation was observed in only 31% of the control group, 38% of the parietal group, and 25% of the cerebellar group.

Between group comparisons of these correlations showed that the stroke patients with parietal and cerebellar lesions maintained coordination between the timing of PV and MA similar to controls. There was no significant group effect (Controls *M* = 0.8, SE = 0.1; Parietal *M* = 0.7, SE = 0.1; Cerebellar *M* = 0.7, SE = 0.1). The relationship between the PV and MA was weaker for the perturbed trials although we found no significant effect of condition and no interaction.

There was a significant effect of condition (*F*_2, 58_ = 3.216, *p* < 0.05) upon the correlation between PD and MA, with baseline scores reliably higher than unperturbed and perturbed trials. We found no significant group and condition interaction. Whilst there was no main group effect (Controls *M* = 0.8, SE = 0.1; Parietal *M* = 0.7, SE = 0.1; Cerebellar *M* = 0.5, 0.1) coordination between PD and the time of MA was weakest for the cerebellar group for the unperturbed trials (*t*_22_ = 1.989, *p* = 0.06).

The correlation between the last velocity peak (TLPV) and TLPA were highest for perturbed scores, and a main condition effect (*F*_2, 58_ = 5.748, *p* < 0.001) was observed. Pairwise comparisons of the Fisher *z* scores for TLPV and TLPA showed a significant difference (*p* < 0.05) between perturbed trials and both the baseline and unperturbed trials. We found no main group effect (Controls *M* = 0.87, SE = 0.1; Parietal *M* = 0.82, SE = 0.1; Cerebellar *M* = 0.82, SE = 0.1).

### Correlation between clinical impairment and reach-to-grasp movement variables (unperturbed trials)

The level of function (FMUL) was significantly correlated (Spearman’s rho) with MD (*r* = −0.73, *p* < 0.01) and TPV (*r* = 0.61, *p* < 0.01). MD was shorter and TPV occurred later in patients with least impairment. FMUL was not correlated with wrist trajectory distance, TMA, amplitude of MA, or trunk movement distance. Age did not correlate with any of the movement variables. FMUL scales for patients were significantly negatively correlated to the time taken to complete 10HPT (*r* = −0.76, *p* < 0.01).

The 10HPT time was positively correlated with MD (*p* < 0.01, *r* = 0.795); the wrist trajectory distance (*p* < 0.01, *r* = 0.772); and negatively correlated with TPV (*p* < 0.01, *r* = −0.474). No correlation was found between 10HPT and TMA; MA; Trunk movement distance or coordination (Pearson’s *r* between TPV and TMA) or age.

## Discussion

The study aimed firstly to identify specific reach-to-grasp coordination impairments associated with either parietal or cerebellar lesions and secondly to compare their movement response to perturbation of the object location. Main findings were that in response to perturbation: (1) parietal participants had a longer wrist path (50° condition only) and a later grasp onset time, compared to cerebellar participants; (2) parietal participants showed a longer RT and MD, a lower PV, earlier% time to PV and more velocity peaks, than controls; and (3) cerebellar participants showed a showed a longer RT and MD, a lower PV and more velocity peaks (10° condition only), than controls.

### Comparison between parietal and cerebellar participants: Unperturbed and baseline trials

Results for unperturbed movements show some similarities between the two patient groups in terms of how they compared to controls. Movement onset was delayed for both groups. In accordance with previous research in heterogeneous and parietal stroke (Michaelsen et al., 2004; Thielmann et al., 2004; Lang et al., 2005; vanVliet and Sheridan, 2007) and cerebellar lesions (Haggard et al., 1994; Bastian and Thach, 1995; Rand et al., 2000; Zackowski et al., 2002; Brandauer et al., 2008; Konczak et al., 2010; Kuper et al., 2011), MD was longer and PV was reduced for parietal and cerebellar participants.

The reasons for these similar findings in the two groups are likely to be different. In parietal participants, the prolonged MD with lower PV likely reflects a greater reliance on feedback (vision and proprioception) for spatial aspects of the movement, due to an impaired ability to localize visual target position, and process sensory information to maintain an estimate of arm state. The longer deceleration phase found in this group (earlier %TPV) compared to control participants allows more time to make use of this feedback to make corrections. The corrections are manifested as an increased number of velocity peaks and cause a longer WPT. Previous work in middle cerebral artery (MCA) stroke (parietal region is supplied by the MCA) (Jeannerod, 1986; vanVliet and Sheridan, 2007) also found a longer deceleration phase compared to controls.

In cerebellar participants however, the prolonged MD was not accompanied by a prolonged deceleration phase compared to controls, and there was not a significant difference in the number of velocity peaks in unperturbed trials. Instead, they exhibited alterations in grasp, where there were multiple aperture peaks and the hand opened earlier. Multiple peaks could be attributed to compensation for inaccurate transport of the hand, as suggested in previous studies where cerebellar participants (Bastian and Thach, 1995; Zackowski et al., 2002) also showed multiple aperture and velocity peaks, and a larger MA, than controls. Alternatively, it could be explained by an impaired ability to adjust relative strength and timing of muscle activations based on internal prediction about the likely outcome of the movement, since Fisher et al. (2006) found that cerebellar participants had errors in target direction and amplitude specification. The early onset of grasp aperture in the cerebellar group, which has been noted before (Haggard et al., 1994; Rand et al., 2000) may be the result of impaired ability to coordinate different effectors in the arm and hand, a function normally performed by the cerebellum.

A previous study (Zackowski et al., 2002) found greater WPT variability and a greater number of velocity peaks in a cerebellar group when compared to controls, which we did not. The contrasting findings may partly reflect differences between the patients in terms of the pathology or symptom severity. Eight out of 10 patients reported in the previous study (Zackowski et al., 2002), suffered from cerebellar atrophy whereas there was no atrophy in our group. Eight of the 10 patients in the previous study also demonstrated moderate or severe ataxia, whereas only 1 of the cerebellar patients here presented with severe coordination/speed deficits (Fugl-Meyer et al., 1975), the remainder having more mild to moderate impairments.

Although correlations between key events were present and have been previously found in cerebellar patients with lesions in the posterior and superior cerebellar artery territory (Kuper et al., 2011), correlational analyses were present in a smaller number of the cerebellar group for all three coupled events compared to other groups, and there was a trend toward a weaker correlation between PD and the time of MA, compared to the parietal and control groups. This echoes previous findings where increased variability of time to MA was noted in cerebellar participants with MA occurring both before and after PD (MA is usually after PD in healthy people) (Zackowski et al., 2002) and higher variability of timing of MA and PV in cerebellar subjects compared to controls (Rand et al., 2000). Two reasons are posited for this: firstly that the cerebellar damage affected the ability to combine transport and grasp into a single functional unit and secondly that it caused impaired parallel processing between shoulder, elbow, and hand (Timmann et al., 1999), which is normally controlled by the cerebellum.

Parietal participants, in contrast, showed similar correlations between time to MA and time to both PV and PD, to controls. Significant correlations between TMA and TPD were also found previously in people with lesions of the areas supplied by the MCA (vanVliet and Sheridan, 2007). One explanation for the fact that parietal participants and cerebellar to some extent, had intact coordination between events, could be that coordination between transport and grasp for RTG may be partially controlled by another area of the brain such as the basal ganglia. The basal ganglia has already been implicated in the control of RTG for managing the sequencing of movements (Fagg and Arbib, 1998). Alternatively, specific structures within the parietal lobe thought to play a key role in coordination, such as the PPC, were perhaps undamaged in this small sample of participants. More rigorous testing and reporting of the lesion size and location with neuroimaging techniques such as fMRI would be needed in a future study to verify this account. The most likely explanation is that in parietal participants impaired coordination was compensated for by moving more slowly to allow more feedback driven control of movements. The delayed grasp onset noted in this group could also be an attempt to maintain synchrony with the delayed transport onset to retain temporal coordination between transport and grasp. However, the grasp adjustment did not extend to the earlier %TMA noted in some previous studies (Lang et al., 2005; Nowak et al., 2007), instead being statistically similar to controls (Jeannerod, 1984; Wallace et al., 1990; vanVliet and Sheridan, 2007).

Stroke patients were expected to compensate for limited arm use with a strategy of trunk recruitment as an additional degree of freedom (Trombly, 1992; Roby-Brami et al., 1997; Michaelsen et al., 2004). Anterior trunk displacement was indeed higher for stroke participants however the difference in the amplitude was not significant. Cirstea and Levin (2000) found that level of motor impairment on the FM scale was correlated with trunk displacement. We found no such correlation. This may be explained by the fact that in their study the task was to point to a target just beyond the reach of the arm, whereas in our study the 35 cm reach distance was within arm’s length, thus requiring less trunk displacement.

### Comparison of parietal and cerebellar group response to location perturbation

Control participants responded to perturbation with an earlier and lower amplitude peak wrist velocity, which is likely due to interruption in initial movement or movement re-organization (Paulignan et al., 1990, 1991a,b). They demonstrated a double peak of grasp aperture as in previous research (Paulignan et al., 1990, 1991a,b) and a second wrist velocity peak (Paulignan et al., 1991b). Correlation between peak wrist velocity and MA was largely preserved for perturbed trials, with MA occurring later. Temporal rescaling in response to perturbation was supported by the significant correlations identified between the timing of both the peak wrist velocity and PD with MA. The last PV also correlated significantly with TLPA.

There were some differences in how controls responded to the respective 10° and 50° perturbations. The 10° perturbation showed a longer MD compared to the 50° perturbation. %TMA also occurred later for perturbed 10° trials than at baseline whereas there was no difference between baseline and 50° perturbations. These differences are most likely due to the greater complexity of motor organization needed for the 10° target position which required more wrist extension to be added to the shoulder flexion/adduction and elbow extension movements of the upper arm used to move to the targets, and more finger extension to open the hand more.

We now turn to consider how patients with right parietal or cerebellar lesions adjust transport-to-grasp when hand transport is perturbed and to verify whether the on-line adjustments necessary to maintain coordination are intact. There were both similarities and differences between the stroke groups in perturbed conditions. Firstly, they were similar in that MD increased during perturbation trials by 13% (0.21 s) and 12% (0.16 s) for parietal and cerebellar patients, respectively. There was a similar number of aperture peaks, in response to perturbation, in both stroke groups and these were comparable to controls. Correlations between the last TMA and both the last TPV and last TPD for both patient groups, were similar to that of controls during perturbed trials.

However, perturbation to both 10° and 50° targets caused significantly more wrist velocity peaks for the parietal group, compared to controls, suggesting a greater reliance on feedback driven control. The 50° perturbation also caused a longer WPT in the parietal group. In perturbed trials, as in baseline and unperturbed trials, the parietal group had a later onset of grasp. These findings could be due to difficulty in localizing visual target position and updating the motor plan for a new target position, since the parietal cortex is known to be involved in these functions (Pisella et al., 2000; Ramnani et al., 2001), so the relocation of the target may have been more difficult for the group with parietal stroke. Parietal participants also slowed movements, with increased aperture peaks, but in addition, increased the number of velocity peaks and had a longer WPT due to difficulties planning the new transport goal and thus relying more on feedback control. The later grasp onset could also reflect planning difficulties, or alternatively could indicate an attempt to maintain synchrony with the delayed transport onset as they appear to retain temporal coordination between transport and grasp better than cerebellar participants. Only 25% trials in the cerebellar group showed correlations between maximum velocity and MA in perturbed trials, compared to 31% of the control group and 38% of the parietal group.

Cerebellar participants presented with early onset of grasp when perturbed. This may demonstrate that the need to compensate for inaccurate transport of the hand overrode the need for the synchrony between start of hand aperture and hand transport. In the perturbed 10° trial condition, the timing of MA (TMA%) was more variable for the cerebellar group, in contrast to controls. Also, although there was no increase in velocity peaks in baseline and unperturbed trials, these appeared in the perturbed 10° trials. This reflects an impaired ability to make rapid adjustments to the perturbation.

### Implications for contribution of parietal and cerebellar regions to reach-to-grasp coordination

The findings confirm the role of parietal cortex in processing sensory information to keep an updated estimate of upper limb state related to visual target position. This was indicated by the longer deceleration phase, with increased use of feedback to guide transport, evidenced by an increased number of velocity peaks and aperture peaks, in people with parietal lesions. This is reinforced by the longer wrist path in response to perturbation, indicating that its normal role in updating the motor plan for a new visual target position, was impaired.

Whereas for the cerebellum, its important role in combining transport and grasp into a single functional unit was shown by the increased variability of the percentage time of MA, the early onset of grasp, and the more impaired temporal coupling between transport and grasp in cerebellar participants. Additionally, the compensatory increased number of aperture peaks seems to support the role of the cerebellum in adjusting relative strength and timing of muscle activations. Lastly, the appearance of increased velocity peaks when cerebellar participants were perturbed, indicates the cerebellum is involved in making rapid adjustments to perturbations. Therefore, our study more clearly differentiates the roles of parietal and cerebellar areas in reach-to-grasp coordination, showing the cerebellum to be more dominant in the temporal coupling, and the parietal area more concerned with using sensation to relate arm and hand state to target position. Although both are involved in responding to location perturbation, the cerebellum adjusts strength and timing of muscle activations, where the parietal cortex relates upper limb state to new target position.

### Correlation between clinical impairment and reach-to-grasp movement variables

Fugl-Meyer Upper limb scores were similar across the two stroke groups. Correlation between stroke participant Fugl-Meyer upper limb scores (Fugl-Meyer et al., 1975) and RTG movement variables indicated that MD was reliably shorter and TPV occurred later in patients with least impairment.

The study had several limitations that should be acknowledged. Ideally, it would have been desirable to compare patients with right hemisphere cerebellar lesions with the group with right-sided parietal lesions. However, it was not possible to recruit sufficient participants with a right hemisphere cerebellar lesion. Secondly, due to resource constraints it was not possible to obtain MRI scans for more accurate localization of lesion. It is recommended that these points be taken into consideration in future studies.

## Conclusion

Whereas there are studies examining the movement deficits of individual patient groups after stroke, it is rare to directly contrast different lesion groups in the same experimental paradigm. This study has demonstrated some contrasting motor deficits in reach-to-grasp following parietal and cerebellar stroke, and different responses of these groups to perturbation of object location. The longer deceleration phase, increased number of velocity peaks and aperture peaks, and longer wrist path in parietal participants, indicates the role of the parietal region in using sensation to relate arm and hand state to target position. Cerebellar participants showed impaired correlation between time of MA and time of PV and PD, increased variability of the percentage time of MA, and early onset of grasp, reflecting the role of the cerebellum in temporal coupling between transport and grasp. The two groups also showed some different responses to perturbation, with parietal participants lengthening the wrist path and the deceleration phase, while cerebellar participants opened the hand earlier.

## Conflict of Interest Statement

The authors declare that the research was conducted in the absence of any commercial or financial relationships that could be construed as a potential conflict of interest.
